# Use of corticosteroids in influenza-associated acute respiratory distress syndrome and severe pneumonia: a systemic review and meta-analysis

**DOI:** 10.1038/s41598-020-59732-7

**Published:** 2020-02-20

**Authors:** Yuqing Zhou, Xiaofang Fu, Xiaoxiao Liu, Chenyang Huang, Guo Tian, Cheng Ding, Jie Wu, Lei Lan, Shigui Yang

**Affiliations:** 1 State Key Laboratory for Diagnosis and Treatment of Infectious Diseases, National Clinical Research Center for Infectious Diseases, Collaborative Innovation Center for Diagnosis and Treatment of Infectious Diseases, The First Affiliated Hospital, College of Medicine, Zhejiang University, Hangzhou, 310003 China; 2 Key Laboratory of Precision Diagnosis and Treatment for Hepatobiliary and Pancreatic Tumor of Zhejiang Province, Hangzhou, 310003 China

**Keywords:** Influenza virus, Drug therapy

## Abstract

Influenza-related severe pneumonia and acute respiratory distress syndrome (ARDS) are severe threats to human health. The objective of this study was to assess the effects of systematic corticosteroid therapy in patients with pneumonia or ARDS. The PubMed, EMBASE, Web of Science and SCOPUS databases were searched up to July, 2019. Nineteen studies including 6637 individuals were identified, and fifteen studies (6427 patients) were included in the meta-analysis of mortality. Eighteen were observational studies and one was a randomized controlled trial (RCT). The meta-analysis results showed that corticosteroid therapy was associated with significantly higher mortality (OR 1.53, 95% CI [1.16, 2.01]) and incidence of nosocomial infection (OR 3.15, 95% CI [1.54, 6.45]). Subgroup analysis showed that among patients with unadjusted estimates, the odds of mortality were higher in patients receiving corticosteroid treatment (OR 1.98, 95% CI [1.23, 3.17]), however, among patients with adjusted estimates, the result showed no statistically significant difference between corticosteroid group and control group (OR 1.31, 95% CI [0.95, 1.80]). Current data do not support the routine use of corticosteroids in patients with influenza severe pneumonia or ARDS. RCTs are needed to provide more robust evidence.

## Introduction

Influenza is a viral infection that attacks the respiratory system. Rapidly progressing viral pneumonia and acute respiratory distress syndrome are pulmonary manifestations that are commonly observed in patients with influenza and are associated with considerable mortality^1–3^, representing a severe threat and imparting a substantial financial burden worldwide^4^.

Individuals with community-acquired pneumonia may benefit from systematic corticosteroid therapy, which may block the inflammatory cascade reaction^5^. Corticosteroids could improve the lung tissue damage induced by influenza pneumonia and decrease the risk of mortality in animal models with influenza infections^6,7^. Many clinicians administer corticosteroids as an anti-inflammatory treatment for patients with severe influenza-related pneumonia to stop disease progression and improve clinical outcomes. A large cohort study of patients admitted to 148 ICUs in Spain found that the frequency of corticosteroid treatment by study period was 34.9% in 2009, 39.6% in 2010, 29% in 2013, and 31.4% in 2014^8^. Recently, some studies have shown that corticosteroids may not be beneficial for patients with severe influenza and may even increase mortality^9–11^. However, there is considerable uncertainty regarding whether patients with influenza-related ARDS or severe pneumonia can benefit from adjuvant corticosteroid therapy.

We aimed to systematically review all experimental and observational studies on corticosteroid use in patients with influenza-related ARDS and severe pneumonia. The effect of corticosteroid treatment on clinical outcomes was investigated.

## Methods

### Study eligibility criteria

This systematic review included studies fulfilling the following inclusion criteria: (a) the studies were RCTs, quasi-experimental studies, or observational studies; (b) patients had confirmed influenza-related pneumonia, ARDS (PaO_2_/FiO_2_ < 300 mmHg); (c) the intervention group used corticosteroids, and the comparison group did not, with no restriction set on the dose or duration of the intervention; and (d) the outcomes were mortality, nosocomial infection, length of stay or other clinical outcomes. A study was excluded if it met any of the following criteria: (a) the study was a review article, conference abstract, case report or case series, case-control study; (b) the majority of included patients were immunocompromised; (c) insufficient data were available; (d) overlapping population; (e) studies with fewer than 20 participants. There were no restrictions on influenza subtype, patient age or study setting. If only some of the individuals included in a study fit the eligibility criteria and these individuals had extractable results corresponding to the objective of this study, then the study was included.

Clinical outcomes including mortality, nosocomial infection, duration of mechanical ventilation, length of stay, time to fever alleviation and clinical stability and viral shedding were evaluated.

### Search strategy and study selection

We comprehensively searched the PubMed, EMBASE, Web of Science and SCOPUS databases from inception to July 2019. The core search terms were defined as those related to influenza-related pneumonia, ARDS, acute respiratory failure and corticosteroid use (for details on the search strategy in EMBASE, refer to Supplementary Table S1). The references of eligible studies were screened, and two authors independently reviewed all citations that met the inclusion criteria. Study selection was performed in 2 stages: first, study title and abstract screening; second, full text examination.

### Data extraction and quality assessment

Outcome data were independently extracted from the included studies by two investigators using a previously piloted standardized pro forma. We obtained the following data: (a) characteristics of studies (design, setting, country, period, methodological details for quality assessment); (b) characteristics of participants (demographics, co-morbid illnesses, disease severity, numbers in each group, influenza virus type); (c) characteristics of interventions (type, dose, timing and duration of corticosteroid use); and (d) outcomes.

The quality of each study was independently assessed by two individuals according to the Cochrane Risk of Bias tool for RCTs and the Newcastle-Ottawa Scale for nonrandomized trials and comparative observational studies. Three domains are assessed on the NOS for observational studies^12^: (1) “selection bias”, (2) “comparability bias”, and (3) “outcome bias”. Disagreements at any stage were resolved through discussion with the other authors until consensus was reached.

### Sensitivity analysis

We performed sensitivity analysis to assess the effect of the study design on clinical outcomes using stratification if the number of studies was sufficient.

### Data analysis

Odds ratios (ORs) and their corresponding 95% confidence intervals (CIs) were generated during the analysis of dichotomous outcome data, and mean differences or standardized mean differences and their corresponding 95% CIs were generated during the analysis of normally distributed continuous data. ORs or hazard ratios (HRs) for adjusted outcome estimates and their corresponding 95% CIs were obtained and are presented in the pooled analyses. Medians and interquartile ranges were generated in the analysis of continuous data that were not normally distributed.

The I² test for inconsistency was used to analyse heterogeneity. If I² > 50%, the heterogeneity across studies was significant, and a random-effects model was used in the meta-analysis; otherwise, a fixed-effects model was used. Subgroup analysis was performed in the following areas where possible: adult population versus child population; seasonal influenza versus outbreak influenza or pandemic influenza; ICU versus inpatient; adjusted estimates versus unadjusted estimates; and corticosteroid dose, timing and duration. All statistical analyses were performed using Cochrane systematic review software Review Manager (RevMan; Version 5.3.5; The Nordic Cochrane Centre, The Cochrane Collaboration, Copenhagen, 2014).

### Main results

**Figure 1:**
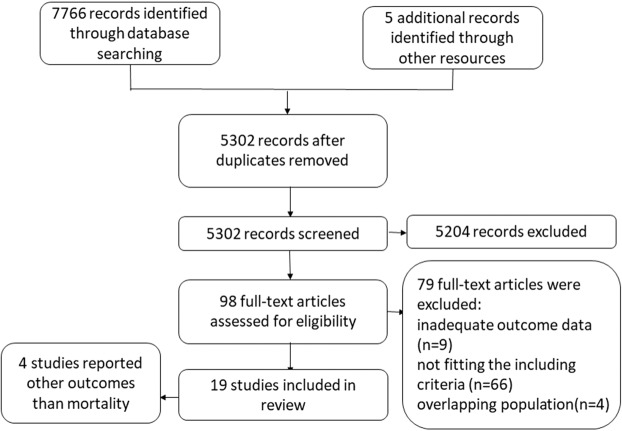
Flow diagram of the article screening process.

A total of 7771 relevant articles were identified during the initial search. After the removal of 2469 duplicates, 5302 articles remained. After screening the titles and abstracts of those articles, 5204 articles were excluded because of irrelevance. Of the 98 full-text articles reviewed, 81 were excluded for various reasons, and 19 articles remained. Details regarding the reasons for the exclusion of these studies are shown in Fig. 1 and Supplementary Table S2. Ultimately, 15 studies were included in the meta-analysis of mortality, while 4 studies reported outcomes other than mortality in association with corticosteroid use.

The characteristics of the participants in the included studies are summarized in Table 1 and Supplementary Table S3. The studies were published between 2010 and 2018. Eighteen of the studies had an observational design, while one had a randomized controlled trial design^13^. Outcome data were reported in 19 studies (6637 individuals, including 2675 in corticosteroid group and 3962 in the non-corticosteroid group), while mortality data were reported in 15 studies (6427 individuals). Eight studies (n = 2558) included only ICU patients. Fourteen studies assessed individuals with H1N1pdm09 virus infection, 1 study assessed individuals with H7N9 virus infection, and 4 study assessed individuals with inter-pandemic influenza virus infection. Eight studies (1956 individuals) had useable data related to patients with ARDS^9,10,14–19^. Fourteen studies (n = 6335) reported mortality associated with adults only.

**Table 1:** Characteristics of the cohorts assessed in the included studies.

Study ID	Design	Setting	CS/no-CS	Corticosteroid	Therapy type and Number
**Studies included in meta-analysis of mortality**					
Brun-Buisson^14^ (France)	Multicenter retrospective analysis	ICU; ARDS; H1N1	83/125	Daily dose: Median (IQR): 270 mg (200–400 mg) hydrocortisone Eq; Timing to initiation from MV, d, Median (IQR): ≤1.0 (0–6.0); Duration, Median (IQR): 11.0 (16.0–20.0)	Prednisone: 4/83, Hydrocortisone: 48/83, Methylpred: 31/83 Antiviral: 204/208 MV: 208/208; ECMO: 53/208
Carrillo-Esper^23^ (Mexico)	Single-center retrospective analysis	ICU; ARDS; H1N1	13/13	Methylpred: 1 mg/kg/d	Antiviral: 26/26 MV: 23/26; Activated protein C and statins: 26/26
Linko^19^ (Finland)	Multicenter prospective cohort	ICU; ARDS; H1N1	46/12	Highest dose, mg, Mean ± SD: 94 ± 43 for methylpred, 214 ± 66 for hydrocortisone; timing, after symptom onset, Median (IQR): 5.0 (2.8–8.3)	MV: 58/58
Rios^24^ (Argentina)	Multicenter prospective cohort study	ICU; Pneumonia; H1N1	75/103	NA	Antiviral: All: 174/178; Survivor: 91/93; Death: 82/85
Sertogullarindan^25^ (Turkey)	Single-center prospective analysis	ICU; Pneumonia; H1N1	7/13	NA	Antiviral therapy: 20/20 Antibiotic: 18/20, Invasive MV: 10/20
Kim^9^ (Korea)	Multicenter retrospective analysis	ICU; ARDS; H1N1	66/70	Dose Eq (pred), mg/d: Median (IQR): 75 (50–81), Timing, d, Median (IQR): after symptom onset, Median (IQR): 5.0 (2.8–8.3)	Antiviral: 136/136
Viasus^20^ (Spain)	Multicenter, prospective cohort study	Inhospital, Pneumonia; H1N1	37/129	17/37 received dose >300 mg/day of hydrocortisone Eq; Duration: Median (IQR): 9.0 (5.0–13.5)	Antiviral: 166/166 Antibiotic: 161/166
Lee^26^ (Singapore, China)	Single-center retrospective cohort study	Inhospital; Pneumonia; Influenza A/B	264/817	NA	NA
Cao^10^ (China)	Multicenter retrospective analysis	Inhospital; Pneumonia; H7N9	204/84	Low to moderate dose 25–150 mg/d, high-dose: 150 mg/d Methylpred; Duration: Median (IQR): 7.0 (4.0–11.3)	Methylpred: 187/204 Dexamethasone: 8/204 Hydrocortisone: 5/204, Antiviral: 285/288; Antibiotic: 261/288 MV: 126/288; ECMO: 36/288
Huang^17^ (Taiwan/China)	Single-center retrospective analysis	Inhospital; Pneumonia, Influenza A/B	29/19	Timing: early (before/within 72 hours of NAIs): 17/29; Duration: short (≤3 days): 4/29; 4 to 13 days: 14/29; ≥14 days: 10/48	Antiviral: 48/48 MV: 39/48 ECMO: 10/48
Moreno^8^ (Spain)	Multicenter prospective cohort study	ICU; Pneumonia, Influenza A/B/C	604/1242	Methylpred Eq, Median (IQR): 80 (60–120) mg; Median duration: 7.0 (5.0–10.0) d	Methylpredn: 578/604 Prednisolone 23/604, Dexamethasone 3/604
Li^15^ (China)	Multicenter retrospective analysis	Inhospital, Pneumonia; H1N1	1055/1086	Daily dose, mg/d, (Eq methylpred), Median (IQR) 80(53.3–160); Duration, Median (IQR): 7.0 (4.0–8.0); Time to initiation from the onset of illness, Median (IQR): 6.0 (4.0–8.0)	Methylpred: 939/1055 Dexamethasone: 85/1055 Hydrocortisone: 21/1055 Prednisolone: 5/1055 Antiviral: 2047/2141 Antibiotic: 2092/2141
Chawla^18^ (India)	Single-center retrospective analysis	ICU; ARDS; H1N1	38/39	Duration, Mean ± SD: 10.6 ± 7.8 days	MV: 36/77(Invasive MV: 19/77); Oseltamivir use: 75–150 mg twice daily
Xi^16^ (China)	Multicenter retrospective analysis	Inhospital; ARDS; H1N1	38/24	Daily dose, mg/d, (Eq methylprednisolone), Median (IQR): 80 (80–160)	NA
Kinikar^27^ (India)	Single-center retrospective analysis	Inhospital/ICUPneumonia; H1N1	21/71	NA	All received antiviral and antibiotic; MV: 20/92
**Studies not included in meta-analysis of mortality but other outcomes**					
Wirz^13^ (Switzerland)	Multicenter RCT	Inhospital, Pneumonia; influenza	11/13	Prednisone (50 mg orally for 7 days)	NA
Chien^21^ (Taiwan/China)	Multicenter retrospective analysis	Inhospital; ARDS; H1N1	21/75	8 patients use hydrocortisone <300 mg/d; 7 patients use Methypred <2 mg/kg/d; 6 patients use high-dose Methypred	Hydrocortisone: 8/21 Methypred: 13/21 Antiviral: 96/96
Kudo^29^ (Japan)	single-center retrospective cohort study	Inhospital; Pneumonia, H1N1	46/12	Dose: Median (IQR): Methylpred dose, 1.0–1.5 mg/kg, 2–4 times/day; Duration, Median: 5.1; Timing, after symptom onset, Median: 2.1 d	Antiviral: 58/58 MV: 0; Antibiotics: CS: 41/46, No-CS: 6/12
Kil^28^ (South Korea)	Single-center retrospective analysis	Inhospital; Pneumonia; H1N1	17/15	Rapid, high-dose (Methylpred, 10 mg/kg per day), and short-term (tapered off within a week)	Antiviral: 30/32 received Oseltamivir within 48 h

Abbreviations: CS, corticosteroid therapy; Eq, equivalent; IQR, interquartile range; SD, standard deviation; ICU, Intensive Care Unit; Methypred, methylprednisolone, MV, mechanical ventilation; ECMO, extracorporeal membrane oxygenation.

The median ages varied from 2.5 to 60.1 years in all patients included. The proportion of male participants was higher than that of females (58.9% versus 41.1%) and range varied from 44.8% to 78.0% (15 studies, 2969 individuals). Obesity (BMI ≥ 30 kg/m^2^) was common (31.7%, 779/2454) in the included studies (7 studies, 2454 individuals), and the proportion of obese individuals ranged from 2.1% to 41.4%. Disease severity at baseline was reported in seven studies and in 4 studies (2166 individuals), the baseline disease severity was higher in individuals in the corticosteroid group than in those in the non-corticosteroid group^19–21^. Methylprednisolone (88.7%, 809/912) was the most common steroid used in the corticosteroid group (4 studies), and the median duration varied from 5.1 to 11.0 days. Almost all patients (97.0%, 5056/5211) received antiviral therapy (ranging from 96.0% to 100%, 12 studies), and 96.5% (2579/2673) of participants received antibiotic therapy (5 studies). The details of the therapies are described in Table 1.

Because all studies included in the meta-analysis of mortality were observational cohort studies, selection bias was inevitable. The risk of bias identified in the 19 included studies is shown in Supplementary Tables S4a,b. The studies’ NOS scores varied from 6 to 9, indicating that the quality of the included studies was high^22^. However, most included studies had substantial comparability bias because we could not adequately adjust for disease severity, and individuals with greater diseases severity tended to use corticosteroids.

### Mortality

#### Overall mortality in the included studies

**Figure 2:**
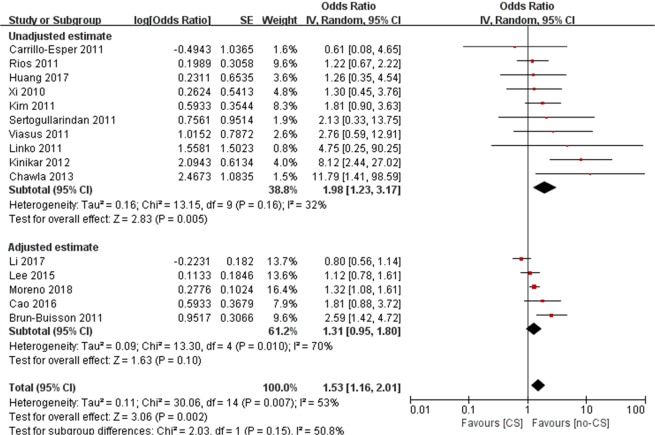
Meta-analysis of studies reporting mortality data. Abbreviations: CI, confidence interval; OR, odds ratio.

Mortality data were reported in 15 studies^8–10,14–20,23–27^. The pooled analysis of the crude results of the 15 included studies (6427 individuals) suggested that those who used corticosteroids had a significantly higher mortality rate (OR 2.30, 95% CI [1.68, 3.16], p < 0.01), and a moderate level of heterogeneity was observed (I² = 66%; Supplementary Fig. S1). Five studies^8,10,14,15,26^ reported adjusted effect estimates of 30-day or inhospital mortality (adjusted OR or adjusted hazard ratio (aHR), Table 2). The pooled analysis of the crude results of ten included studies and five adjusted effect estimates suggested that those who used corticosteroids had a significantly higher mortality rate (OR 1.53, 95% CI [1.16, 2.01], I^2^ = 53%, p < 0.002, Fig. 2). The subgroup analysis of unadjusted effect estimates showed a similar result (OR 1.98, 95% CI [1.23, 3.17], I^2^ = 32%, p = 0.005). However, the subgroup analysis of the five adjusted estimates showed no association between mortality and corticosteroid use (HR 1.31, 95% CI [0.95, 1.80], I^2^ = 70%, p = 0.1). The test for subgroup differences between adjusted and unadjusted mortality was not statistically significant (p = 0.06). There was no clear indication of publication bias in the funnel plot analysis (Supplementary Fig. S2).

**Table 2:** Summary of studies reporting adjusted mortality outcome data.

Study, Year	Outcome	Mortality/Patients		Risk of Mortality (95% CI)		Variable (s) for Adjusted Risk
		CS	No-CS	Unadjusted	Adjusted	
Cao^10^	30 d mortality	81/204	11/84	OR, 4.37 (2.19–8.74)	HR, 1.81 (0.88–3.74)	60 years old older, invasive ventilation, NAI antivirals, platelet counts, were used to adjust for the effects of the corticosteroids in COX regression analysis
Brun-Buisson^14^	Inhospital mortality	28/83	21/125	HR, 2.39 (1.32–4.31)	HR, 2.59 (1.42–4.73)	Immunosuppression, vasopressor use, SAPS III were used to adjust in COX regression analysis
Moreno^8^	ICU mortality	166/604	234/1242	OR, 1.63 (1.30–2.05)	HR, 1.32 (1.08–1.60)	Age, APACHE II score, Gap ICU, Number quadrants infiltrates in chest X-ray, LDH, CPK, Acute kidney failure, CRRT, AKIN classification, Serum urea, Serum procalcitonin, MV, non-invasive MV failure, Chronic heart disease, No chronic heart disease, pregnancy, HIV/AIDS, Neuromuscular disease, Autoimmune disease, Immunosuppression, VAP were used in Cox Regression analysis after propensity score matching analysis
Lee^26^	Inhospital mortality	50/264	87/817	OR, 1.96 (1.34–2.87)	HR, 1.12 (0.78–1.61)	Age per 20 years, Male sex, H1N1pdm09, Bacterial superinfection, NAI treatment, Statin use were used to in Cox regression models
Li^15^	Inhospital PneumoniaH1N1	232/1055	74/1086	OR, 3.86 (2.92–5.09)	HR, 0.80 (0.56–1.15)	Adjusted for underlying comorbidities, baseline disease severity, NAI treatment, time duration from disease onset to hospitalization in the Cox regression model
Kim^9^	90-d mortality	38/66	25/70	OR, 2.44 (1.22–4.87)	OR, 1.80 (0.69–4.69)	Adjusted for age, SOFA score, and lymphocyte count, the propensity score by backward step-wise logistic regression

Abbreviations: CI, confidence interval; CS, corticosteroid; OR, odds ratio; HR, hazard ratio; IQR, interquartile range; MV, mechanical ventilation; SD, standard deviation; NAI: Nueraminidase inhibitor; APACHE, Acute Physiology and Chronic Health Evaluation; ICU, intensive care unit; MV, mechanical ventilation; SAPS, Simplified Acute Physiology Score; SOFA, Sequential Organ Failure Assessment; CRP, C-reactive protein;LDH, lactate dehydrogenase; WBC, white blood cell.

#### Mortality in ARDS

**Figure 3:**
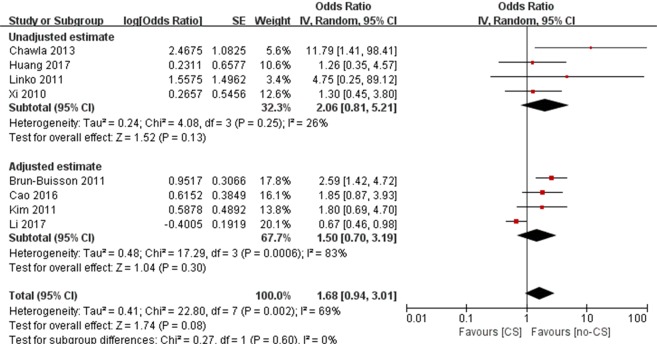
Meta-analysis of studies mortality data in patients with ARDS. Abbreviations: CI, confidence interval; OR, odds ratio.

Eight studies reported the mortality of patients with ARDS due to influenza (n = 1956, Supplementary Table S5**)**. Meta-analysis of unadjusted and adjusted estimates suggested that the difference in mortality between the corticosteroid and control groups was not statistically significant (OR 1.68, 95% CI [0.94, 3.01], I^2^ = 69%, p = 0.08, Fig. 3). Li *et al*. reported that corticosteroid use was associated with a decreased risk of mortality in patients with ARDS (PaO_2_/FiO_2_ < 300 mmHg) (aHR 0.67, 95% CI [0.46, 0.98])^15^. However, Brun-Buisson *et al*.^14^ and Chawla *et al*.^18^ suggested that the risk of mortality was higher in corticosteroid group in the patients with ARDS. Cao *et al*. reported that a low-to-moderate dose of corticosteroids had no statistically significant association with the risk of mortality in patients with ARDS (OR 1.69, 95% CI [0.78, 3.64]), whereas a high dose was associated with greater mortality (OR 2.89, 95% CI [1.10, 7.56])^10^. The other four studies^9,16,17,19^ showed that corticosteroid use had no association with mortality (Fig. 3).

#### Sensitivity analysis

Considering the different management strategies for paediatric and adult patients, one study investigating paediatric patients was excluded^27^. The pooled analysis of 14 studies reporting adult individuals revealed a significant increase in the odds of mortality with corticosteroid use, with moderate statistical heterogeneity (OR 2.17 [1.59, 2.96], I^2^ = 65%).

#### Subgroup analysis

A subgroup analysis according to pure ICU patients (8 studies, 2558 individuals) and mixed patients (7 studies, 3878 individuals including both ICU and wards patients) was conducted. For the pure ICU subgroup, we found that corticosteroids were associated with an increased risk of mortality (OR, 1.71 [1.41, 2.06]) with low statistical heterogeneity (I^2^ = 7%), and a similar result was found for mixed patient groups (OR 3.14 [2.58, 3.83], I^2^ = 62%)(Supplementary Fig. S3).

Three studies (n = 3473) have reported the results of mortality excluding patients who had potential indications (e.g., asthma, COPD exacerbation, pregnancy/post-partum, shock or immunosuppressive conditions) for corticosteroid treatment that may have skewed the results^8,14,15^. A pooled analysis of these three studies showed no statistically significant association between corticosteroid use and mortality (OR 1.37, 95% CI [0.86, 2.18]), with a high level of heterogeneity (I² = 77%, P = 0.01).

Thirteen studies included adult patients with A/H1N1, A/H3N2 or B influenza. The pooled analysis of these studies found corticosteroid use to be associated with greater odds of mortality (OR 2.03, 95% CI [1.47, 2.79], with a moderate level of heterogeneity (I^2^ = 63%).

The number of studies was insufficient to perform subgroup analysis according to the various reported regimens. Two studies compared early versus later/no corticosteroid treatment; one defined early treatment as within three days of mechanical ventilation^14^, and the result suggested that early treatment with corticosteroids was associated with greater mortality (aHR 3.42, 95% CI [1.73, 6.75]); the other defined early treatment as within 72 h of NAIs^17^, and the result showed no statistically significant difference in mortality between patients receiving corticosteroids within 72 h of NAIs and those who did.

Three studies categorized corticosteroid dose as low/low-to-moderate and high^10,15^. A large retrospective cohort study reported that a low-to-moderate dose (25–150 mg/d methylprednisolone or equivalent) was associated with a decreased risk of mortality in patients with PaO_2_/FiO_2_ < 300 mmHg (aHR 0.51, 95% CI [0.33–0.78]), whereas in patients with PaO_2_/FiO_2_ ≥ 300 mmHg (aHR 0.88, 95% CI [0.56, 1.39]), a low-to-moderate dose was associated with greater mortality (aHR 3.70, 95% CI [1.20–11.34])^15^. A high dose (>150 mg/d methylprednisolone or equivalent) showed no benefit in all patients^15^. A retrospective Chinese cohort study of 288 people with influenza A H7N9 virus infection suggested that compared to the controls, the mortality risk in patients receiving low-to-moderate doses of corticosteroids (25–150 mg/d methylprednisolone or equivalent), was not significantly different (aHR 1.64, 95% CI [0.79, 3.39]), whereas in participants treated with high-dose corticosteroids (defined as > 150 mg/d methylprednisolone or equivalent), the mortality risk was significantly greater (aHR 3.05, 95% CI [1.28, 7.25])^10^. Another study reporting the result of 62 patients with acute respiratory failure due to influenza showed no statistically significant difference between low dose and high dose corticosteroid therapy (8/19 versus 7/19, p > 0.05)^16^.

Two of the included studies reported outcomes related to children, but only one reported the risk of mortality (OR 8.12, 95% CI [2.44, 27.02]) related to corticosteroid use^27^. However, in that study, all children who received corticosteroids had ARDS, while the patients in the non-corticosteroid group had less severe disease conditions. Another retrospective cohort study of children with pneumonia caused by the 2009 H1N1 influenza virus only reported length of hospital stay and duration of fever^28^ and found a shorter length of stay and duration of fever in corticosteroid group (Table 3).

**Table 3:** Summary of studies reporting clinical outcomes other than mortality.

Outcome	Median/Mean	Study Year	CS treatment	No-CS treatment	P value
Length of ICU stay; day	Median (IQR)	Moreno^8^	10.0 (5.0–19.0)	8.0 (5.0–18.0)	P = 0.05
		Linko^19^	18.0 (13.0–20.0)	4.0 (3.0–5.0)	P < 0.001
		Brun-Buisson^14^	22.0 (13.0–39.0)	17.0 (11.0–30.0)	P = 0.11
Length of hospital stay, day	Mean (95% CI)	Huang^17^	29.0 (23.3–34.7)	25.7 (14.1–37.0)	P = 0.59
	Median (IQR)	Linko^19^	24.0 (14.0–37.0)	15.0 (8.0–25.0)	P = 0.06
	Median (range)	Kudo^29^	8.2 (5.0–14.0)	7.7 (3.0–14.0)	P = 0.607
	Mean ± SD	Wirz^13^	9.2 ± 9.4	10.4 ± 8.0	P > 0.05
		Kil^28^	6.4 ± 1.1	8.5 ± 7.0	P > 0.05
Duration of MV, days	Median (IQR)	Linko^19^	10.0 (5.0–13.0)	0 (0–2.0)	P = 0.001
		Moreno^8^	8.0 (3.0–17.0)	8.0 (3.0–16.0)	P = 0.96
		Brun-Buisson^14^	17.0 (10.0–29.0)	13.0 (8.0–24.0)	P = 0.07
Time to clinical stability, days	Median (IQR)	Viasus^20^	4.0 (2.0–6.0)	2.5 (1.0–5.)	P < 0.05
		Wirz^13^	4.0 (1.4–7.0)	5.0 (3.0–10.4)	P < 0.05
Time to fever alleviation, days	Median (range)	Kudo^29^	1.5 (0.4–6.3)	1.48 (0.4–7.0)	P = 0.967
	Mean ± SD	Kil^28^	2.1 ± 0.8	5.8 ± 4.8	P = 0.009
Viral shedding, days	Median (IQR)	Cao^10^	14.0 (12.0–17.0)	12.0 (11.0–15.0)	p < 0.05

Abbreviations: CS, corticosteroid therapy; ICU, Intensive Care Unit; MV, mechanical ventilation.

#### Nosocomial infection

**Figure 4:**
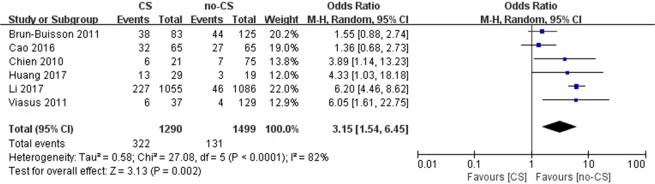
Meta-analysis of studies reporting nosocomial infection data. CI, confidence interval; OR, odds ratio.

Six studies reported an association between corticosteroid use and nosocomial infection. In four of these studies, corticosteroid use was associated with an increased risk of developing a nosocomial infection^15,17,20,21^, while the remaining two studies did not show a statistically significantly increased odds of developing infection^10,14^. Overall, the pooled results revealed that the odds of nosocomial infection were significantly higher in patients who were administered corticosteroids than in those who were not (OR 3.15, 95% CI [1.54, 6.45], p < 0.0001), but a high level of heterogeneity was observed (I² = 82%) (Fig. 4).

Three studies reported the common pathogens isolated from patients with nosocomial infection. One study reported that the most common bacteria isolated was *Acinetobacter baumannii* (28.2%)^17^. In a study of 2141 patients with severe influenza pneumonia^15^, 245 patients had nosocomial infection, and the most commonly isolated pathogens were *Acinetobacter baumannii* (35.0%), *Pseudomonas aeruginosa* (13.5%), and *Staphylococcus aureus* (11.0%), while in another cohort study of 1846 patients with severe influenza pneumonia^8^, *Streptococcus pneumoniae* (49.1%), *Pseudomonas aeruginosa* (10.1%), and *Staphylococcus aureus* (7.5%) were the most frequently isolated microorganisms.

#### Length of stay and length of MV

Seven studies reported length of stay according to corticosteroid use; all were unadjusted for disease severity (Table 3). Six studies found no statistically significant difference between the groups. One study^19^ showed a longer length of ICU stay associated with corticosteroid use, while the total length of hospital stays was not significantly different between the groups. Notably, one of the five studies analysed the duration of hospital stay in people with influenza pneumonia treated with corticosteroid versus those receiving placebo, and found no significant difference between the groups (adjusted difference −2.24 days, 95% CI [−9.61, 5.12])^13^.

Linko *et al*.^19^ reported a longer duration of mechanical ventilation in the group treated with corticosteroid therapy while Brun-Buisson *et al*.^14^ and Moreno *et al*.^8^ found no statistically significant difference between the groups. (Table 3).

#### Time to fever alleviation, time to clinical stability and viral shedding

Two studies reported the time to fever alleviation according to corticosteroid use^28,29^. Kudo *et al*.^29^ found no statistically significant difference between the groups, while another cohort study of children^28^ with severe influenza pneumonia reported a shorter time to fever alleviation. Notably, two studies found a shorter time to clinical stability in the corticosteroid group. The study of influenza A/H7N9 found a significantly longer duration of viral shedding associated with corticosteroid treatment^10^. The details of these outcomes are described in Table 3.

## Discussion

The overall findings of this meta-analysis indicated that patients with pneumonia or acute respiratory distress syndrome who were administered corticosteroids had significantly higher mortality and incidence of nosocomial infection but the use of corticosteroids did not influence the length of hospital stay.

Our studies suggested a deleterious effect of steroids on mortality and nosocomial infection. Several factors need to be accounted for in interpreting these findings. First, most studies did not adjust the clinical outcomes for potential confounding factors. Clinically, more severe cases tended to be treated with corticosteroids, which may obscure the real value of this treatment regarding mortality^30,31^. Therefore, in this study, we preferred the use of adjusted estimates of the effect to minimize potential confounding between the treatment groups. However, five studies reported adjusted estimates of mortality, and their inclusion in the meta-analysis still revealed a higher odds of mortality related to steroids use. Good evidences indicated that secondary bacterial pneumonia is an important cause of mortality related to influenza^32,33^. Therefore, increasing risk of nosocomial infection due to corticosteroid treatment may partly account for the potential harm from corticosteroid use. Two included studies^8,15^ found that secondary bacterial pneumonia such as due to *Acinetobacter baumannii*, *Pseudomonas aeruginosa*, *Streptococcus pneumoniae*, *Staphylococcus aureus* or invasive fungal infection, were more common in corticosteroid-treated patients. Several studies showed that prolonged viral shedding and delayed viral clearance were noted in corticosteroid-treated patients^10,34^, whereas slower clearance of virus loads was associated with higher mortality in patients with ARDS due to H1N1pdm09 virus infection^35^. Thus, prolonged viral shedding and delayed viral clearance may also contribute to higher mortality.

Second, most of the included observational studies did not explain why some patients received systemic corticosteroid therapy and others did not. The initial intentions of corticosteroid therapy were unclear (was it used as a rescue therapy or due to COPD/asthma exacerbation or due to pneumonia/ARDS?). Different indication may easily confound the effect of the corticosteroid. Some evidences supported the use of corticosteroids for asthma or COPD or septic shock in the context of influenza infection^36–38^. In order to minimize the influences of different indications, subgroup analysis of the mortality in three studies (n = 3347) was performed after excluding patients receiving corticosteroids as rescue therapy or due to COPD/asthma exacerbation, and found no statistically significant difference between the steroid therapy groups and control groups and the heterogeneity was high (I² = 77%). However, the high level of statistical heterogeneity may result in unstable estimates of the meta-analysis. Therefore, well-designed clinical trials should be conducted to decrease the heterogeneity of patients and to provide more robust evidence.

The results from clinical studies of corticosteroid therapy in patients with influenza are conflicting. Many studies have shown a significant association between corticosteroid treatment and mortality in patients with influenza; however, several studies have reported that corticosteroids can provide benefits to patients under certain conditions^15,28,39,40^. An RCT^13^ included in this review noted an association between adjuvant corticosteroid therapy (50 mg of prednisone given orally for 7 days) and decreased time to clinical stability. Low-to-moderate doses of corticosteroids are beneficial in people with hypoxia ((PaO_2_/FiO_2_) <300 mmHg), whereas high doses of corticosteroids showed no benefit in this group; however, low-to-moderate doses of corticosteroids may increase the 60-day mortality rate in those with PaO_2_/FiO_2_ > 300 mmHg^15^. Kil *et al*.^28^ reported that rapid (methylprednisolone, 10 mg/kg/d) and short-term (tapered off within a week) corticosteroid treatment for children with severe pneumonia halted clinical exacerbation and possibly prevented progression to ARDS. However, in another study, compared with no treatment, administration (steroid therapy was initiated at a median daily dose equivalent to 270 (IQR, 200–400) mg of hydrocortisone, and a median duration of 11 (IQR, 6–20) days within the first 3 days of MV was more strongly associated with an increased risk of death, whereas when administration was beyond the first 3 days of MV, the association was no longer significant^14^. Considering the findings of the aforementioned studies, the condition of the patients’ respiratory system and the dose, timing and duration of corticosteroids could be contributing factors that affect the effects of corticosteroids.

Several recent systematic reviews and meta-analyses concluded that corticosteroid therapy is significantly associated with mortality^41–43^. However, in these studies, there were no special limitations on subject inclusion criteria, which means that the patients were very diverse. Additionally, there was no subgroup analysis for these patients under different disease conditions. Compared to patients in those previous studies, we focused only on patients with pneumonia or ARDS, which is more specific and makes the outcomes more targeted. Our study observed a different outcome according to corticosteroid use in patients with ARDS due to influenza.

This study has some limitations, including the lack of sufficient data on the dose, duration, timing and rationales of corticosteroid administration and the timing and duration of antiviral therapy. In addition, only one study included in this meta-analysis was an RCT, and 18 were observational in nature. Thus, it is possible that selection bias or comparability bias could have affected the quality of the analysed evidence. There is insufficient evidence in this meta-analysis to make a firm determination about the effectiveness of corticosteroids for people with influenza-related pneumonia or ARDS. The small number of included studies and the small number of patients in the included studies might also make the effect size of some outcome indicators insufficient, and we were unable to analyse the effect of some factors on the outcome indicators by meta-regression or subgroup analysis.

## Conclusion

Current data do not support the routine use of corticosteroids in patients with influenza pneumonia or ARDS. However, the data assessed in this meta-analysis were extracted from 18 observational studies and only one RCT; therefore, the limitations associated with study design are important to consider. There is a need for more robust evidence on the role of corticosteroids in the treatment of influenza-related ARDS and severe pneumonia before a firm recommendation for clinical practice can be made.

## Supplementary information

Supplementary materials.
