# Supplementary material for: Use of corticosteroids in influenza-associated acute respiratory distress syndrome and severe pneumonia: a systemic review and meta-analysis
Source: Sci Rep. 2020 Feb 20;10:3044. doi: 10.1038/s41598-020-59732-7 (PMC7033254; doi:10.1038/s41598-020-59732-7)
Supplement: Supplementary file 1 — Supplementary materials. [file 41598_2020_59732_MOESM1_ESM.pdf]

Use of corticosteroids in influenza-associated acute respiratory distress syndrome and severe pneumonia: a systemic review and meta-analysis

Yuqing Zhou[1], Xiaofang Fu[1], Xiaoxiao Liu[1], Chenyang Huang[1], Guo Tian[1,2], Cheng Ding[1], Jie Wu[1], Lei Lan[1], Shigui Yang[1]

[1]State Key Laboratory for Diagnosis and Treatment of Infectious Diseases, Collaborative Innovation Center for Diagnosis and Treatment of Infectious Diseases, National Clinical Research Center for Infectious Diseases, The First Affiliated Hospital, College of Medicine, Zhejiang University, Hangzhou 310003, China

[2]Key Laboratory of Precision Diagnosis and Treatment for Hepatobiliary and Pancreatic Tumor of Zhejiang Province, Hangzhou 310003, China

1, Supplementary Figure S1: Meta-analysis of studies reporting crude mortality data.

	CS		no-CS			Odds Ratio
Study or Subgroup	Events	Total	Events	Total	Weight	M-H, Random, 95% CI
Brun-Buisson 2011	28	83	21	125	9.0%	2.52 [1.31, 4.85]
Cao 2016	81	204	11	84	8.6%	4.37 [2.19, 8.74]
Carrillo-Esper 2011	2	13	3	13	2.2%	0.61 [0.08, 4.41]
Chawla 2013	9	38	1	39	1.9%	11.79 [1.41, 98.42]
Huang 2017	9	29	5	19	4.3%	1.26 [0.35, 4.57]
Kim 2011	31	66	23	70	8.6%	1.81 [0.90, 3.63]
Kinikar 2012	9	21	6	71	4.7%	8.13 [2.44, 27.05]
Lee 2015	50	264	87	817	12.1%	1.96 [1.34, 2.87]
Li 2017	232	1055	74	1086	13.2%	3.86 [2.92, 5.09]
Linko 2011	7	46	0	12	1.1%	4.75 [0.25, 89.12]
Moreno 2018	166	604	234	1242	13.6%	1.63 [1.30, 2.05]
Rios 2011	38	75	47	103	9.6%	1.22 [0.67, 2.22]
Sertogullarindan 2011	4	7	5	13	2.4%	2.13 [0.33, 13.81]
Viasus 2011	3	37	4	129	3.3%	2.76 [0.59, 12.92]
Xi 2010	15	38	8	24	5.5%	1.30 [0.45, 3.80]
**Total (95% CI)**		**2580**		**3847**	**100.0%**	**2.30 [1.68, 3.16]**
Total events	684		529			

Heterogeneity: Tau² = 0.18; Chi² = 41.28, df = 14 (P = 0.0002); I² = 66%
Test for overall effect: Z = 5.18 (P < 0.00001)

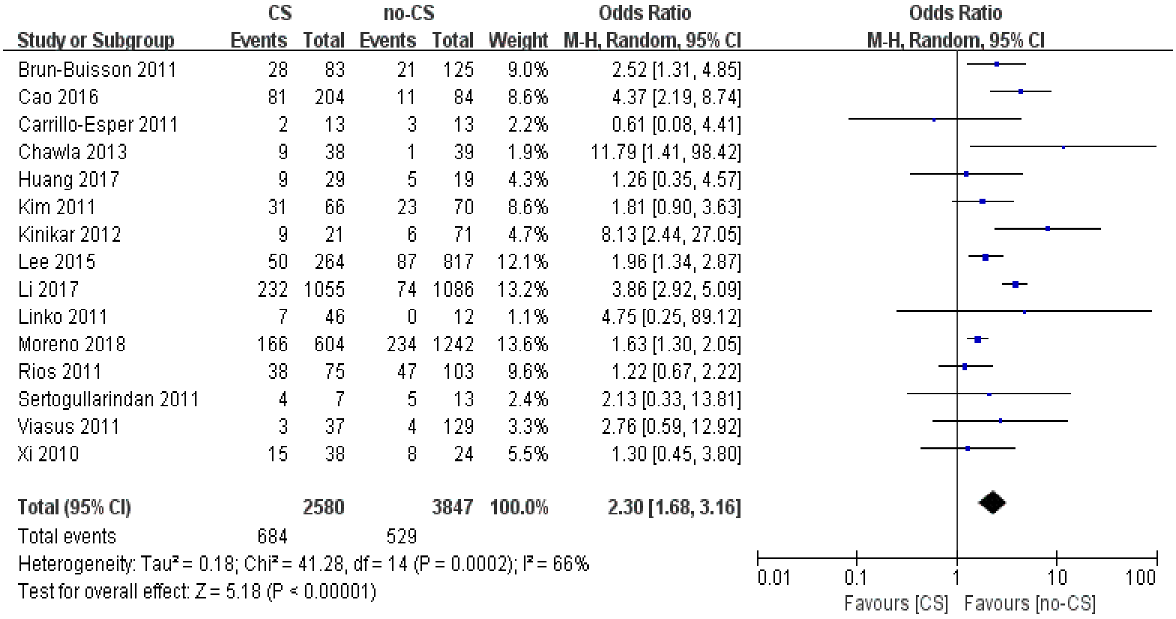

Abbreviations: CI, confidence interval; OR, odds ratio

2, Supplementary Figure S2: Funnel plot of studies reporting mortality

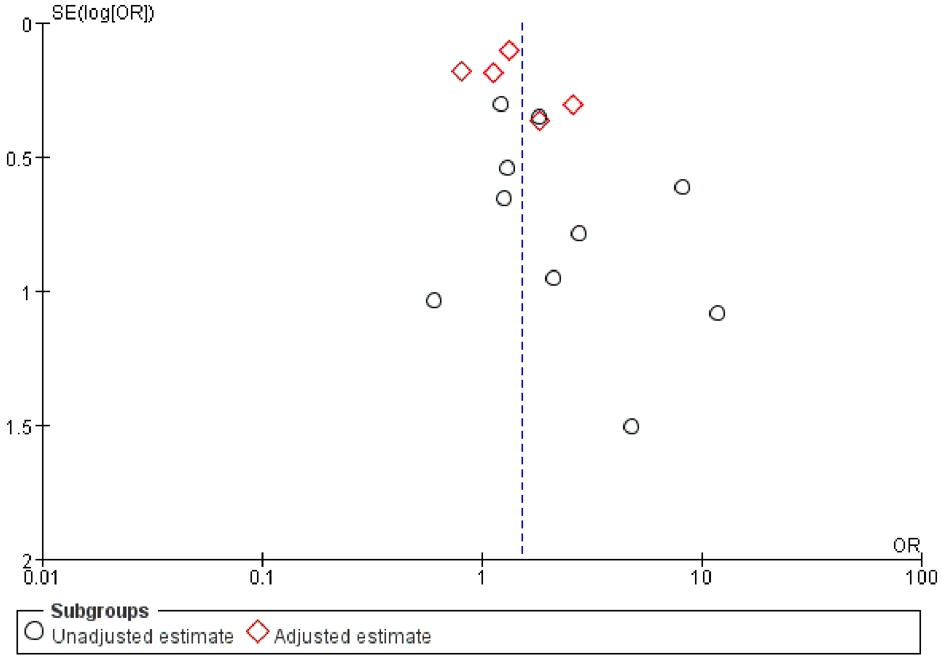

3，Supplementary Figure S3: Subgroup analysis of pure ICU patients and mixed patients

Study or Subgroup	CS Events	CS Total	no-CS Events	no-CS Total	Weight	Odds Ratio M-H, Fixed, 95% CI
**Pure ICU patients**						
Brun-Buisson 2011	28	83	21	125	4.1%	2.52 [1.31, 4.85]
Carrillo-Esper 2011	2	13	3	13	0.9%	0.61 [0.08, 4.41]
Chawla 2013	9	38	1	39	0.3%	11.79 [1.41, 98.42]
Kim 2011	31	66	23	70	4.3%	1.81 [0.90, 3.63]
Linko 2011	7	46	0	12	0.2%	4.75 [0.25, 89.12]
Moreno 2018	166	604	234	1242	40.7%	1.63 [1.30, 2.05]
Rios 2011	38	75	47	103	7.2%	1.22 [0.67, 2.22]
Sertogullarindan 2011	4	7	5	13	0.5%	2.13 [0.33, 13.81]
**Subtotal (95% CI)**		**932**		**1617**	**58.2%**	**1.71 [1.41, 2.06]**
Total events	285		334			
Heterogeneity: Chi² = 7.50, df = 7 (P = 0.38); I² = 7%						
Test for overall effect: Z = 5.52 (P < 0.00001)						
**Mixed patients**						
Cao 2016	81	204	11	84	3.4%	4.37 [2.19, 8.74]
Huang 2017	9	29	5	19	1.5%	1.26 [0.35, 4.57]
Kinikar 2012	9	21	6	71	0.6%	8.13 [2.44, 27.05]
Lee 2015	50	264	87	817	12.6%	1.96 [1.34, 2.87]
Li 2017	232	1055	74	1086	20.8%	3.86 [2.92, 5.09]
Viasus 2011	3	37	4	129	0.6%	2.76 [0.59, 12.92]
Xi 2010	15	38	8	24	2.2%	1.30 [0.45, 3.80]
**Subtotal (95% CI)**		**1648**		**2230**	**41.8%**	**3.14 [2.58, 3.83]**
Total events	399		195			
Heterogeneity: Chi² = 15.84, df = 6 (P = 0.01); I² = 62%						
Test for overall effect: Z = 11.33 (P < 0.00001)						
**Total (95% CI)**		**2580**		**3847**	**100.0%**	**2.31 [2.01, 2.64]**
Total events	684		529			
Heterogeneity: Chi² = 41.28, df = 14 (P = 0.0002); I² = 66%						
Test for overall effect: Z = 12.08 (P < 0.00001)						
Test for subgroup differences: Chi² = 18.98, df = 1 (P < 0.0001), I² = 94.7%						

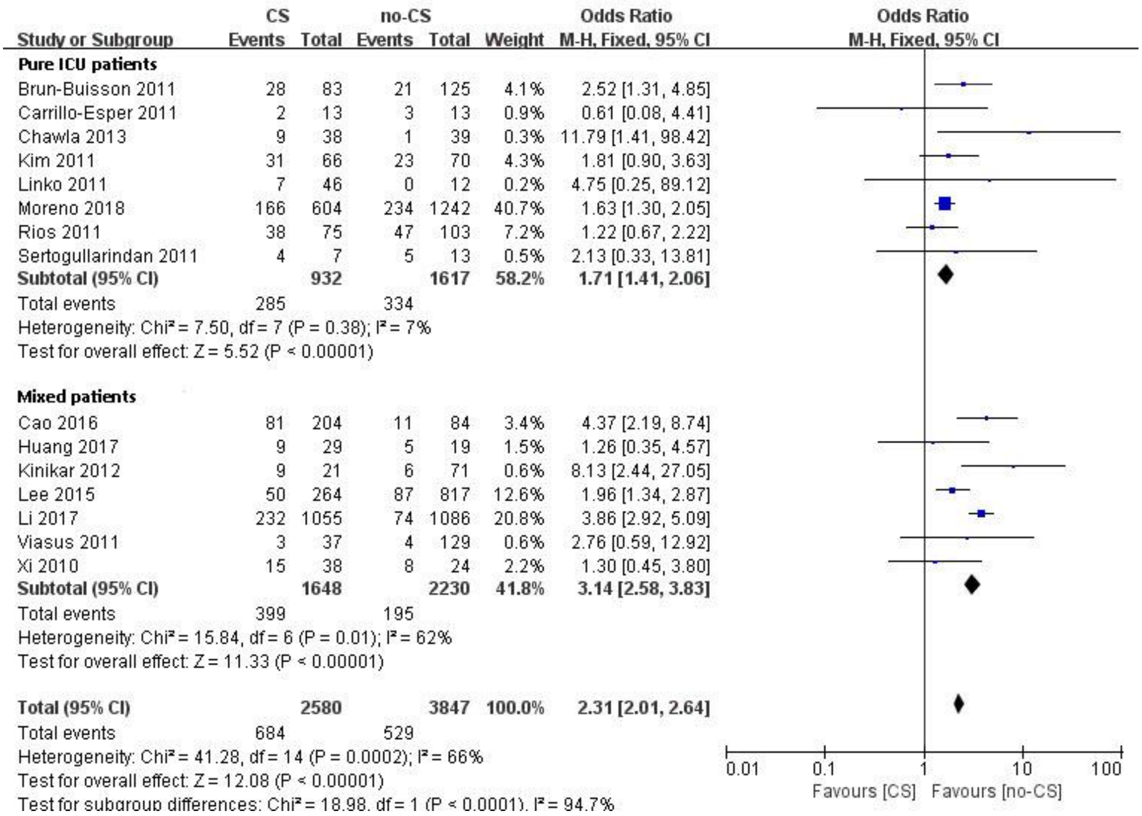

4, Supplementary Table S1: Search strategy on EMBASE

No.	Query
#16	#13 AND #14 AND #15
#15	#1 OR #10
#14	#2 OR #3 OR #4 OR #5 OR #6 OR #7 OR #11
#13	#8 OR #9 OR #12
#12	steroid:ti,ab,kw OR steroid*:ti,ab,kw OR corticosteroid:ti,ab,kw OR corticosteroid*:ti,ab,kw OR corticoid:ti,ab,kw OR corticoid*:ti,ab,kw OR adrenocorticosteroid:ti,ab,kw OR hydrocortisone:ti,ab,kw OR prednisolone:ti,ab,kw OR cortisone:ti,ab,kw OR glucocorticoid:ti,ab,kw OR 'adrenal cortex hormone':ti,ab,kw OR methylprednisolone:ti,ab,kw
#11	pneumonia:ti,ab,kw OR pneumonia*:ti,ab,kw OR pneumonitis:ti,ab,kw OR pneumonitides:ti,ab,kw OR 'pulmonary inflammation*':ti,ab,kw OR 'pulmonary inflammation':ti,ab,kw OR 'shock lung':ti,ab,kw OR ards:ti,ab,kw OR ardss:ti,ab,kw OR 'acute respiratory distress syndrome':ti,ab,kw OR 'adult respiratory distress syndrome':ti,ab,kw OR 'acute chest syndrome':ti,ab,kw OR 'respiratory distress syndrome':ti,ab,kw OR 'acute lung injury' OR 'acute respiratory failure' OR ARF OR ALI
#10	'influenzavirus a':ti,ab,kw OR 'influenzavirus b':ti,ab,kw OR 'influenzavirus c':ti,ab,kw OR influenza:ti,ab,kw OR influenza*:ti,ab,kw OR flu:ti,ab,kw OR flus:ti,ab,kw OR grippe*:ti,ab,kw OR h1n1:ti,ab,kw OR h2n2:ti,ab,kw OR h3n2:ti,ab,kw OR h5n1:ti,ab,kw OR h7n9:ti,ab,kw OR h7n1:ti,ab,kw OR h7n2:ti,ab,kw OR h7n3:ti,ab,kw OR h7n7:ti,ab,kw OR h9n2:ti,ab,kw
#9	'corticosteroid'/exp
#8	'steroid'/exp
#7	'acute respiratory failure'/exp
#6	'acute lung injury'/exp
#5	'respiratory distress syndrome'/exp
#4	'acute chest syndrome'/exp
#3	'adult respiratory distress syndrome'/exp
#2	'pneumonia'/exp
#1	'influenza'/exp

5, Supplementary Table S2: Excluded studies

Study ID	Reason for exclusion
Diaz[1] 2012	Overlapping cohort, superseded by later analysis
Perez-Padilla[2] 2009	Case series
Singh[3] 2017	No useable data relating to patient with pneumonia or ARDS
Martin-Loeches[4] 2011	Overlapping population
Sheu[5] 2017	Conference abstract
Marin-Corral[6] 2018	No useable data relating to patient with pneumonia or ARDS
Yeh[7] 2018	No data on clinical outcomes
Jung[8] 2011	Overlapping population
Wang[9] 2018	Overlapping population
Mady[10] 2012	No useable data relating to patient with pneumon ia or ARDS
Balaganesakumar[11] 2013	No useable data relating to patient with pneu monia or ARDS
Liem[12] 2009	Only 84% patients with pneumonia
Delaney[13] 2016	No useable data relating to patient with pneumonia or ARDS
Patel[14] 2013	Only 68% patients with pneumonia

References:

1 Diaz, E. *et al.* Corticosteroid therapy in patients with primary viral pneumonia due to pandemic (H1N1) 2009 influenza. *Journal of Infection* **64**, 311-318, doi:10.1016/j.jinf.2011.12.010 (2012).

2 Perez-Padilla, R. *et al.* Pneumonia and respiratory failure from swine-origin influenza A (H1N1) in Mexico. *New England Journal of Medicine* **361**, 680-689, doi:10.1056/NEJMoa0904252 (2009).

3 Singh, A. *et al.* Descriptive Analysis of Mortality Predictors in H1n1 Influenza in South Indian Patients. *Infectious disorders drug targets* **17**, 106-115, doi:10.2174/1871526517666170407155558 (2017).

4 Martin-Loeches, I. *et al.* Use of early corticosteroid therapy on ICU admission in patients affected by severe pandemic (H1N1)v influenza A infection. *Intensive care medicine* **37**, 272-283, doi:10.1007/s00134-010-2078-z (2011).

5 Sheu, C. C., Chang, W. A., Tsai, M. J., Tsai, J. R. & Chong, I. W. Early Corticosteroid Treatment In Patients With Influenza-Associated Acute Respiratory Distress Syndrome. *American journal of respiratory and critical care medicine* **195** (2017).

6 Marin-Corral, J. *et al.* Patients with influenza A (H1N1)pdm09 admitted to the ICU. Impact of the recommendations of the SEMICYUC. *Medicina intensiva* **42**, 473-481, doi:10.1016/j.medin.2018.02.002 (2018).

7 Yeh, C. Y. *et al.* Clinical outcomes and prognostic factors of patients with severe influenza receiving intravenous peramivir salvage therapy in intensive care units. *Journal of microbiology, immunology, and infection = Wei mian yu gan ran za zhi* **51**, 697-704, doi:10.1016/j.jmii.2017.06.001 (2018).

8 Jung, J. Y. *et al.* Acute kidney injury in critically ill patients with pandemic influenza A pneumonia 2009 in Korea: A multicenter study. *Journal of critical care* **26**, 577-585,

doi:10.1016/j.jcrc.2011.02.012 (2011).

9 Wang, Y. *et al.* Factors Associated With Prolonged Viral Shedding in Patients With Avian Influenza A(H7N9) Virus Infection. *Journal of Infectious Diseases* **217**, 1708-1717, doi:10.1093/infdis/jiy115 (2018).

10 Mady, A. *et al.* Clinical experience with severe 2009 H1N1 influenza in the intensive care unit at King Saud Medical City, Saudi Arabia. *Journal of infection and public health* **5**, 52-56, doi:10.1016/j.jiph.2011.10.005 (2012).

11 Brun-Buisson, C., Richard, J. C., Mercat, A., Thiebaut, A. C. & Brochard, L. Early corticosteroids in severe influenza A/H1N1 pneumonia and acute respiratory distress syndrome. *American journal of respiratory and critical care medicine* **183**, 1200-1206, doi:10.1164/rccm.201101-0135OC (2011).

12 Liem, N. T. *et al.* Clinical features of human influenza a (H5N1) infection in vietnam: 2004-2006. *Clinical Infectious Diseases* **48**, 1639-1646, doi:10.1086/599031 (2009).

13 Delaney, J. W. *et al.* The influence of corticosteroid treatment on the outcome of influenza A(H1N1pdm09)-related critical illness. *Critical Care* **20**, doi:10.1186/s13054-016-1230-8 (2016).

14 Patel, K. K. *et al.* Clinical Outcome of Novel H1N1 (Swine Flu)-Infected Patients During 2009 Pandemic at Tertiary Referral Hospital in Western India. *Journal of global infectious diseases* **5**, 93-97, doi:10.4103/0974-777x.116868 (2013).

6, Supplementary Table S3: Characteristics of the cohorts assessed in the included studies

Study, Year	Participants					Disease severity score	Outcome(s)
	CS/no-CS	Age	Male Sex	BMI	Comorbidities		
Studies included in meta-analysis of mortality							
Brun-Buisson 2011 (France)	83/125	Median age (IQR), All: 47.0 (35.0-55.0) CS: 49.0 (34.0-56.0); no-CS: 45.0 (35.0-55.0)	All: 105/208 CS: 36/83; no-CS: 69/125	Median (IQR), All: 28.0 (24.0-33.0) CS: 29.0 (24.0-33.0) no-CS: 27.0 (23.0-33.0) BMI >=30: All: 86/208, CS: 34/83 no-CS: 52/125	Immunodepression: 41/208 in all patients, 23/125 in no-CS group,18/82 in CS group; Diabetes: 27/208, 20/125, 7/83; Pregnancy or postpartum: 14/208, 9/125, 5/83; Chronic liver disease, alcohol abuse: 14/208, 8/125, 6/83; Chronic renal failure :3/208, 2/125, 1/83; Neuromuscular disease: 6/208,4/125, 2/83	SAPS III, Median (IQR): All: 52.0 (44.0-64.0); CS: 51.0 (44.0-61.0); no-CS: 53.0 (46.0-66.0); p>0.05	Mortality, ICU-acquired infection, duration of MV, length of ICU stay, Ventilator-free days
Carrillo-Esper 2011 (Mexico)	13/13	Mean ± SD, All: 52.3 ± 15.5; Survivor: 53.6 ±14.9; Death: 46.6 ± 18.4	All: 16/26 Survivor: 13/21 Death: 3/5	Mean ± SD; All: 28.8 ± 4.1 Survivor: 28.8 ± 4.2 Death: 28.7 ± 4.2	Diabites:6/26; 9/26 high blood pressure; cardiovascular disease: 4/26;	APACHE II, Mean ± SD, 13.1±6.4; SOFA, Mean ±SD, 6.4±3.4	Mortality
Xi 2010	38/24	Not reported	Not reported	Not reported	Not reported	Not reported	Mortality
Cao 2016 (China)	204/84	Median age (IQR): All: 58.0 (45.0-68.0) Low-dose CS: 59.0 (49.3-68.0) high-dose CS: 57.5 (46.5-68.8) no- CS: 55.0 (38.3-67.0)	low-dose CS: 118/168 high-dose CS: 28/36 no-CS: 55/84	Not reported	Hypertension: 74/168, 13/26, 26/84; Heart disease:18/168, 2/36, 11/84; Diabetes:36/168, 6/36, 9/84; COPD or Asthma: 6/168, 5/26, 3/84; Chronic renal insufficiency: 5/168, 1/36, 3/84; Immunosuppressed: 0/168, 0/36, 3/84; Pregnancy: 3/168 1/26, 2/84	Not reported	30d,60d-Mortality; Nosocomial infections; Viral shedding

Kim 2011 (Korea)	66/70	Not reported	Not reported	Not reported	Not reported	Not reported	14d, 30d,90d-Mortality
Rios 2011 (Argentina)	75/103	Mean ± SD,<br>All: 44.0 ± 15.0<br>Survivor: 45.0 ± 16.0<br>Death: 43.0 ± 15.0	All: 98/178<br>Survivor: 49/93<br>Death: 49/85	All: 28 ± 8<br>Survivor: 28 ± 6 Death: 29 ± 10<br>BMI>=30:<br>All: 46/178,<br>Survivor:17/93<br>Death: 29/85	BMI>=30: 46/178 in all patients, 17/93 in survived group and 29/85 in deceased group; BMI:28 ± 8 in all patients, 28 ± 6 in survivors, 29 ± 10 in deceased; COPD 28/178 in all patients 16/93 in survivors, 12/85 in deceased,Asthma 10/178 in all patients 6/93 in survivors, 4/85 in deceased; diabetes, 16/178, Immunosupression,29/178; Pregnancy 16/178,	APACHE II, Mean±SD,<br>All: 18.0 ± 7.0<br>Survivor:17.0 ± 6.0<br>Death: 20.0 ± 7.0<br>SOFA,<br>All: 4.0 (5.0-8.0)<br>Survivor: 3.0 (5.0-7.0)<br>Death: 6.0 (4.0-8.0)	Mortality
Lee 2015 (China, Singapore)	264/817	Not reported	Not reported	Not reported	Not reported	NA	Mortality
Linko 2011 (Finland)	46/12	Median (IQR),<br>CS:<br>51.0 (40.0-56.0);<br>no-CS:<br>50.0 (44.0-57.0)	CS: 29/46<br>no-CS: 8/12	BMI >35:<br>CS: 11/46<br>No-CS: 4/12	Chronic obstructive pulmonary disease: 1/12 in no-CS group, 3/46 in CS group; Ischemic cardiac disease: 1/12, 6/46; Pregnancy 0/12, 1/46;<br>BMI >35, 4/12, 11/46	SAPS II, Median (IQR),<br>CS: 32.0 (22.0-37.0)<br>no-CS: 26.0 (17.0-30.0), p<0.05; SOFA,<br>no-CS:4.0 (0-7.0)<br>CS:7.0(4.0-90), p<0.05	ICU mortality, length of stay, Hospital Mortality, length of stay, Length of MV
Li 2017 (China)	1055/ 1086	Median: 34.4	CS: 530/1055<br>no-CS: 565/1086	Not reported	Immunosuppressive conditions: 34/1055 in CS, 15/1084 in no-CS; Hypertension: 158/1055, 168/1086; Diabetes:78/1055, 90/1086; Cardiovascular disease: 56/1055, 75/1086; COPD or Asthma: 81/1055, 63/1086; Chronic renal disease:36/1055, 38/1086	Not reported	Mortality, Nosocomial infection

Huang 2017 (Taiwan/ China)	29/19	Median: 60.1	30/48	BMI > 30: 1/48	Immunocompromised: 16/48; Malignancy: 9/48; Chronic lung disease: 13/48; Diabetes: 22/48	Not reported	Mortality, Length of Stay
Sertogullari-ndan 2011 (Turkey)	7/13	Mean±SD (range), 36.0±2.8 (15.0-72.0)	10/20	BMI>30: 1/20	Cardiac disorder:4/20; COPD: 2/20; Renal diseases: 2/20	Not reported	Mortality
Viasus 2011 (Spain)	37/129	Median (IQR),<br>CS: 44.0 (35.0-53.0);<br>no-CS: 35.0 (28.0-47.0)	CS: 15/37<br>no-CS: 61/129	Not reported	Chronic pulmonary disease:22/129 in no-CS group, 17/27 in CS group; Diabetes:8/129 in no-CS group, 5/37 inn CS group; chronic heart disease:8/129, 3/37	high risk PSI classes,<br>CS: 8/37<br>no-CS: 8/129<br>P <0.05	In-hospital mortality, Time to clinical stability, Nosocomial infection
Moreno 2018 (Spain)	604/1242	Median (IQR),<br>CS: 53.0 (41.0-62.0)<br>no-CS: 51.0 (39.0-61.0)	All: 1096/1846<br>CS: 357/604<br>no-CS: 739/1242	BMI>30:<br>All: 608/1846<br>CS:221/604<br>no-CS: 387/1242<br>P<0.05	Asthma: 79/604 in CS, 75/1242 in-no-CS P<0.05; COPD: 154/604 in CS, 178/1242 in no-CS P<0.05; chronic heart disease: 56/604, 126/1242, P>0.05; chronic renal disease: 53/604 in-CS, 98/1242 in no-CS P>0.05; Hematological disease 65/604 in CS, 68/1242 in no-CS group, p=0.001	APACHE II score, median (IQR),<br>CS: 15 (10-20)<br>no-CS: 14 (10-19)<br>p=0.004;<br>SOFA, median (IQR)<br>CS: 5 (4-8),<br>no-CS:5(3-8), p=0.33	ICU mortality, ICU length of stay, length of MV
Chawla 2013(India)	38/39	Mean± SD (IQR), 40.9 ±13.5 (10.0-72.0)	44/77	Not reported	Diabetes:15/77; Hypertension: 14/77 Asthma:13/77 Coronary Artery Disease: 5/7 , Hypothyroidism 4/77 immunosuppressants:6/77, peripartum:3/77	Not reported	Mortality
Kinikar 2012(India)	21/71	Median (IQR),<br>2.5 (1.3-6.0)	43/92	Not reported	Asthma:4/71	Not reported	Mortality

Studies not included in meta-analysis of mortality but other outcomes							
Kudo 2012 (Japan)	46/12	Median (IQR), 8.0 (0-71.0)	26/58	NA	Asthma:18/58	Not reported	Hours to alleviation of fever after admission; Hospitalization days
Wirz 2016 (Switzerland)	11/13	NA	NA	NA	NA	NA	Time to clinical stability, length of stay
Kil 2011 (South Korea)	17/15	Mean±SD, CS: 6.6 ± 1.5; no-CS:7.8 ± 3.4	CS:13/17 no-CS: 12/15	NA	NA	NA	Duration of fever, length of stay
Chien 2010 (Taiwan/ China)	21/75	Median (IQR), 18 (0.7-73.0)	Male sex: 61/96	BMI>30: 22/96	obesity:22/96; Diabetes: 9/96; Neurodevelopment diseases:12/96; Chronic pulmonary disease:11/96; cardiovascular disease:9/96; chronic renal insufficiency 4/96; immunosuppresion 4/96	SOFA score CS vs no-CS, $p < 0.05$	Nosocomial infection

Abbreviations: APACHE, Acute Physiology and Chronic Health Evaluation; COPD, chronic observation pulmonary disease; CS, corticosteroid therapy; Eq, equivalent; ICU, intensive care unit; IQR, interquartile range; methypred, methylprednisolone, MV, mechanical ventilation; PSI, pneumonia severity index; SAPS, Simplified Acute Physiology Score; SD, standard deviation; SOFA, Sequential Organ Failure Assessment.

7, Supplementary Table S4a: Risk of bias in the included observational studies, as determined using the Newcastle-Ottawa Scale

Study	A. Selection				B. Comparability of cohorts	C. Outcome		
	Represent-activeness of exposed cohort	Selection of non-exposure	Ascertainment of exposure	Outcome not present at start		Assessment of exposure	Follow-up long enough?	Adequacy of follow-up
Brun-Buisson 2011	*	*	*	*	**	*		*
Cao 2016	*	*	*	*	**	*	*	*
Moreno 2018	*	*	*	*	**	*		*
Li 2017	*	*	*	*	**	*	*	*
Kim 2011	*	*	*	*	**	*	*	*
Lee 2015	*	*	*	*	*	*	*	*
Viasus 2011	*	*	*	*	**	*	*	*
Rios 2011	*	*	*	*	**	*	*	*
Carrillo-Esper 2011	*	*	*	*		*		*
Linko 2011	*	*	*	*		*		*
Huang 2017	*	*	*	*		*		*
Sertogullari-ndan 2011	*	*	*	*		*		*
Xi 2010	*	*	*	*		*		*
Chawla 2013	*	*	*	*		*		*
Kinikar 2012	*	*	*	*		*		*
Kudo 2012	*	*	*	*		*		*
Kil 2011	*	*	*	*		*		*
Chien 2010	*	*	*	*		*		*

Supplementary Table S4b: Risk of bias in the included RCTs, as determined using Cochrane Risk of Bias tool

Study	Random sequence generation	Allocation concealment	Blinding of participants and personnel	Blinding of outcome assessors	Incomplete outcome data	Selective outcome reporting	Other bias
Wirz 2016	LOW Restricted randomization process	LOW Allocation concealed	LOW Double blind	LOW Investigators who analyzed the data were masked to treatment allocation.	HIGH Outcomes were assessed from a very much smaller "randomly selected subsample" of total participants	UNCLEAR Trial protocol not available	UNCLEAR. Original cluster-randomization process might have been OK but these outcomes was assessed in a small "randomly selected subsample" of individuals.

8, Supplementary Table S5: Mortality of ARDS in included studies

Study ID	Setting	Participants number	Event/ total CS	Event/ total no-CS	Adjusted estimate
Brun-Buisson 2011	ICU; ARDS; H1N1	All (n=208)	28/83	21/125	aHR,2.59 (1.42-4.37)
Li 2017	Inhospital; ARDS; H1N1	All (n=1160)	215/760	62/400	aHR, 0.67 (0.46-0.98)
		Low-to-moderate dose vs no-CS (n = 836)	99/436	62/400	aHR, 0.49 (0.32-0.77)
		High-dose vs no-CS (n= 712)	111/312	62/400	aHR, 0.88 (0.56-1.39)
Cao 2016	Inhospital; ARDS; H7N9	All (n=207)			aHR, 1.85 (0.87-3.93)
		Low-to-moderate dose vs no-CS (n = 176)			aHR, 1.69 (0.78-3.64);
		High-dose vs no-CS (n = 76)			aHR, 2.89 (1.10-7.56)
Kim 2011	ICU; ARDS; H1N1	All (n=136)	38/66	25/70	aOR,1.80 (0.69-4.70)
Xi 2010	Inhospital; ARDS; H1N1	All(n=62)	15/38	8/24	
		Low-to-moderate dose vs no-CS (n = 43)	8/19	8/24	
		High dose vs no-CS (n =43)	7/19	8/24	
Huang 2017	Inhospital; ARDS; Type A/B	All (n=48)	9/29	5/19	
Chawla 2013	ICU; ARDS; H1N1	All (n=77)	9/38	1/39	
Linko 2011	ICU; ARDS; H1N1	All (n=58)	7/46	0/12	
